# Annotations

**Published:** 1902-06-14

**Authors:** 


### Annotations.

### What to do with the Feeble-minded.

At the special centres for the instruction of the mentally deficient established by the London School Board there are now nearly 2,500 such children in attendance, and no one can doubt that a great deal of good is thus being done. Some of them, in fact, improve so much that it is found possible to pass them on into the ordinary schools, while, on the other hand, some show so little receptivity that they have to be excluded as only fit for an asylum. The rest, however, continue in the special classes until they are passed out into the world, and it becomes a matter of extreme interest to ascertain what then becomes of them. There is no doubt that a fair number take their place, a low place, in the industrial army. But with a large number life is far less uneventful. Turned out into the world the boys, weak in intellect and unable to appreciate the consequences of much that they do, yet impulsive and unable to hold back in the presence of temptation, become hooligans, criminals, paupers, and chronic drunkards, while the girls quickly, horribly quickly, become mothers or drift on to the streets. Thus it is that, notwithstanding all that is being done to improve their condition, the feeble-minded remain the principal source from which the criminal classes are recruited, and the question is seriously being discussed whether the number and working capacity of those who are returned to the ranks of labour is any proper reward to society for the pains taken in educating the feeble-minded, and whether in the case of those who are sufficiently feeble in mind to require special education, it would not be a better plan to continue to care for them through life, society being repaid for the expense so incurred by the diminution of crime and pauperism in the present generation and by the diminution of imbecility in generations yet unborn.

### Security of Tenure of Office for Sanitary Officials.

We can hardly wonder at the anxiety felt by medical officers of health and sanitary inspectors to obtain greater security of tenure of office than they possess at present. The fact that, except in London and Scotland, these officials hold their appointments subject to annual re-election, has been shown again and again to be a great hindrance to the efficient performance of their duty. It would indeed have been difficult to devise any more effectual means of damping the ardour of a medical officer of health than the existing plan of leaving his livelihood at the mercy of those whose shortcomings he has to bring to book. From year end to year end medical officers of health are engaged in a constant war against what may be termed landlords' neglect, and under existing conditions of human nature it seems impossible for them to do their duty without exciting the opposition and in many cases the enmity of those on whose good will their continuance in office depends. Take the simple and daily recurring case of the dwellings of the poor. It is the sanitary inspector, backed up by the medical officer of health, who stands between the poor man and damp walls, leaky roofs, defective drains, foul water, and all other like things which make for ill health. It is the medical officer who has to condemn houses as unfit for human habitation, and to apply for "closing orders" by which they are spoiled as rent-producing properties. Can we wonder then that medical officers often find their position intolerable. There can be no doubt that if the sanitary administration of the country is to be carried out with proper energy and vigour some such Bill as that now before Parliament, for providing security of tenure of office to medical officers of health and sanitary inspectors, ought to become law without delay.

### Fire and the Architects.

The calamitous fire in the City by which ten persons lost their lives has drawn attention in a striking and dramatic manner to the unfitness of many modern buildings for the industries which are carried on within them. The immediate effect of this catastrophe has been to raise a cry of indignation at the defective appliances with which the fire brigade has to work; but, after all, whether the assertions made in this regard be true or not, the primary fault lies, not with the fire brigade but with the architects, by whom such dangerous death-traps are designed, and with the authorities by whom the plans are passed. There would be something ludicrous, if its results were not so horrible, in the disproportion between the immense sums of money which those who would erect great buildings in main thoroughfares are made to pay for purely decorative purposes, and the small sums spent in a perfunctory manner upon little trumpery iron ladders leading, by way of more or less inaccessible trap doors, on to the "leads," for protection in case of fire. To the architects, millions of money, the whole aspect of our towns, and the safety of our workers are entrusted, and yet so tied down are architects by tradition, so enslaved are they by certain preconceived ideas as to what is beautiful, that, casting on one side the eternal truth that beauty lies in fitness, they fill our streets with the monstrosities we see on every side, and, lest their elevations should be spoiled, refuse to make the buildings they design even safe for those who work within them. Architects, above all things, object to what they call excrescences. For years the struggle has gone on about the drains, and now it will have to be seriously undertaken about the fire escapes. Except for the objections raised by architects and municipal authorities there would be but little difficulty in rendering escape from fire a comparatively simple matter in most buildings. In the newer flats this is already done. The true way out of every burning building is by the windows, and we hold that if the surveyors by whom all plans have to be passed would insist on this being recognised, architects would soon be compelled to evolve a style of City architecture in which decorative effect, instead of being obtained by plastering buildings over with scores of useless columns and shafts and mouldings, would be gained by means of light and elegant iron balustrades, providing a ready means of escape from fire and rendering such catastrophies as that of Queen Victoria Street impossible.

